# Verminotic pneumonia in South American fur seal (*Arctocephalus australis*) in Southern Brazil

**DOI:** 10.1017/S0031182022001494

**Published:** 2023-02

**Authors:** Yasmin Daoualibi, Renata F. Moreira, Marcele B. Bandinelli, Joanna V. Z. Echenique, Paulo G. C. Wagner, João F. Soares, Saulo P. Pavarini

**Affiliations:** 1 Setor de Patologia Veterinária, Departamento de Patologia Clínica Veterinária, Faculdade de Veterinária, Universidade Federal do Rio Grande do Sul (UFRGS), Av. Bento Gonçalves 9090, Porto Alegre, RS 91540-000, Brazil; 2 Laboratório de Protozoologia e Rickettsioses Vetoriais, Faculdade de Veterinária, UFRGS, Porto Alegre, Brazil; 3 Instituto Brasileiro do Meio Ambiente e dos Recursos Naturais Renováveis – IBAMA, Porto Alegre, Brazil

**Keywords:** *Arctocephalus australis*, *ITS-2*, metastrongylids, *Parafilaroides*, pinnipeds

## Abstract

Verminotic pneumonia caused by *Parafilaroides* spp. nematodes is an underreported disease in beached South American fur seals, with scant literature available on the characteristics of parafilaroidiasis, the nematode itself, as well as its occurrence in pinnipeds in Brazil. The present work aims to identify, describe and detail the histological features of the infection and molecular characteristics of verminotic pneumonia in the South American fur seal. Twenty-six specimens of *Arctocephalus australis*, found dead on the northern coast of Rio Grande do Sul in 2021, were analysed. These animals were identified and submitted to necropsy and histology. For the molecular identification of metastrongylids, lung fragments were subjected to DNA extraction, polymerase chain reaction targeting the *Internal transcribed spacer 2 (ITS-2)* gene and subsequent sequencing. In total, 12 animals presented with parasites in the lung parenchyma on histological evaluation, and only 1 showed a granulomatous lung lesion at necropsy. Microscopically, the nematodes were found mainly in the alveoli, associated with little or no inflammatory response, and they had morphological characteristics compatible with metastrongylids. Six *ITS-2* gene quality sequences were obtained; after comparative analysis *via* BLAST, they showed similarity with sequences obtained from *Parafilaroides* sp. Therefore, verminotic pneumonia caused by *Parafilaroides* represents an important differential diagnosis of lung disease in South American fur seals found on the northern coast of Rio Grande do Sul.

## Graphical abstract

**Figure:**
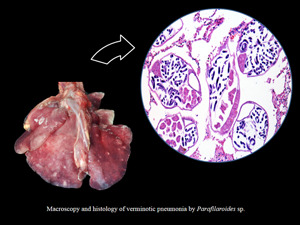

## Introduction

Verminotic pneumonia caused by metastrongylids *Parafilaroides* spp. (Metastrongyloidea: Filaroididae) and *Otostrongylus circumlitus* (Metastrongyloidea: Crenosomatidae) is frequently reported in pinnipeds in different parts of the world (Onderka, 1989; Dailey, 2001, 2009; Siebert *et al*., 2007; Reisfeld *et al*., 2019). While the nematode *O. circumlitus* affects different hosts, the genus *Parafilaroides* has several species that cause lung disease in pinnipeds from different regions (Dailey, 2006, 2009; Jacobus *et al*., 2016; Reisfeld *et al*., 2019; Echenique *et al*., 2020). The parasitic cycle of both genera is not well known, but experimental studies involving *Parafilaroides decorus* and *O. circumlitus* have concluded that fish preyed on by pinnipeds, respectively as opaleye (*Giretta nigricans*) (Dailey, 1970) and turbot (*Psetta maxima*) (Lehnert *et al*., 2010), serve an important role as intermediate hosts. The great diversity of parasitic species from the same genus that affect pinnipeds is probably related to the affinity of each parasite to its respective intermediate and definitive hosts; thus, fur seals of the same species and from the same region, which feed on prey from similar habitats, usually have the same parasitic species (Wells and Clark, 2019).

In fur seals, verminotic pneumonias are caused predominantly by *Parafilaroides* spp. and, to date, there is only evidence of an Antarctic fur seal with *O. circumlitus* pneumonia (Measures, 2001; Dailey, 2009; Jacobus *et al*., 2016; Seguel *et al*., 2018; Reisfeld *et al*., 2019; Echenique *et al*., 2020). *Parafilaroides* spp. comprise 7 species of nematodes distributed around the world (Measures, 2001; Dailey, 2006, 2009; Jacobus *et al*., 2016; Rhyan *et al*., 2018; Reisfeld *et al*., 2019; Echenique *et al*., 2020); of these, only *Parafilaroides hydrurgae* and *Parafilaroides normani* have been reported from the Southern Hemisphere in seals (*Hydrurga leptonyx*) and fur seals (Measures, 2001; Dailey, 2009; Jacobus *et al*., 2016; Echenique *et al*., 2020), respectively. The fur seal was infected by *P. normani*, which was a specimen of *Arctocephalus australis* and presented occurrence at Cassino beach, located on the Southern coast of Rio Grande do Sul in Brazil (Echenique *et al*., 2020). This work aims to identify and quantify the occurrence of pneumonia concomitant with the presence of helminths detected by polymerase chain reaction (PCR) and histology, in South American fur seals found dead on the northern coast of Rio Grande do Sul, as well as to characterize the macroscopic and histological lesions caused by them.

## Material and methods

During the period between autumn and spring 2019–2020, weekly monitoring was carried out on the northern coast of Rio Grande do Sul between the beaches of Tramandaí (30°00′20.6″S 50°07′54.8″W) and the end of Palmares do Sul (30°28′40.4″S 50°19′18.9″W) in partnership with Instituto Brasileiro do Meio Ambiente e dos Recursos Naturais Renováveis (IBAMA; SISBIO: 77472-1; SISBIO: 61987). Twenty-six South American fur seals classified as codes 2 and 3, according to the decomposition classification, were identified and analysed as per the protocol of Geraci and Lounsbury (2005). The age of the animals was determined by the total length of the animals (Ponce de Léon, 1983; Katz *et al*., 2012), while body condition was measured using the adipose tissue of the xiphoid process region and then classified as bad, moderate or good (Geraci and Lounsbury, 2005; Katz *et al*., 2012). Necropsy was performed in a conventional manner (Geraci and Lounsbury, 2005) and fragments of different organs were collected for histological analysis, fixed in 10% buffered formalin and processed routinely. For each animal, 4 fragments were collected from different lung lobes, selected by macroscopic alterations or, if absent, randomly, for histological analysis, totalling 104 fragments.

Two lung fragments from each animal were collected, frozen at −20°C and pooled and subjected to DNA extraction using the commercial Pure Link^®^ Genomic DNA Mini Kit (Thermo Fisher Scientific, Waltham, MA, USA), according to the manufacturer's recommendations. For the PCR, primers (5′GCAGACGCTTAGAGTGGTGAAA3′/R 5′ACTCGCCGTTACTAAGGGAATC3′) flanked in the adjacent genes 5.8S and 28S were used, targeting the *ITS-2* gene according to Lenerth *et al*. (2010). The PCR products were subjected to electrophoresis in 1.5% agarose gel and later visualized in a light-emitting diode transilluminator.

Samples that were equally positive on PCR and histology were selected for purification and subsequent sequencing. Amplicons were purified with the Invitrogen^TM^ PureLink^TM^ Quick PCR Purification Kit (Thermo Fisher Scientific), as per the manufacturer's recommendations and sequenced on an automated sequencer (Sanger, Cambridgeshire, UK). The generated sequences were submitted to BLAST^®^ analysis (Altschul *et al*., 1990) to determine similarity.

The partial sequences of the present study were aligned with another 12 corresponding sequences from Metastrongylids and a sequence from *Dictyocaulus filaria* (as an outgroup) available from GenBank^®^ using Clustal/W v.1.8.1 (Thompson *et al*., 1994). A maximum-likelihood phylogenetic tree with 260 informative sites was generated using the Hasegawa–Kishino–Yano + G substitution model. This was created with the aid of Mega 10 software, using 100 bootstrap replicas (Kumar *et al*., 2018), in accordance with the lowest Bayesian information criterion score. An identity matrix with 452 informational sites was calculated with the BioEdit software using partial sequences of *ITS-2* from *Parafilaroides* species deposited in GenBank and those found in this study (Table 1).

**Table 1.** Identity matrix of the *Parafilaroides* sp. sequence for the present study (1 at 6) and isolates of *Parafilaroides* deposited in GenBank^®^

Sequences	[ON059340] *Parafilaroides* sp. (1)	[ON059341] *Parafilaroides* sp. (2)	[ON059342] *Parafilaroides* sp. (3)	[ON059343] *Parafilaroides* sp. (4)	[ON059344] *Parafilaroides* sp. (5)	[ON059345] *Parafilaroides* sp. (6)	[KP402084] *Parafilaroides* sp.	[KP402085] *Parafilaroides* sp.	[LT984655] *Parafilaroides decorus*	[FJ787304] *Parafilaroides gymnurus*
*Parafilaroides* sp. (1)	ID									
*Parafilaroides* sp. (2)	0.99	ID								
*Parafilaroides* sp. (3)	0.995	0.995	ID							
*Parafilaroides* sp. (4)	0.988	0.988	0.992	ID						
*Parafilaroides* sp. (5)	0.995	0.995	1	0.992	ID					
*Parafilaroides* sp. (6)	0.99	0.99	0.995	0.995	0.995	ID				
[KP402084] *Parafilaroides* sp.	0.995	0.995	1	0.992	1	0.995	ID			
[KP402085] *Parafilaroides* sp.	0.99	0.997	0.995	0.988	0.995	0.99	0.995	ID		
[LT984655] *Parafilaroides decorus*	0.897	0.893	0.897	0.895	0.897	0.897	0.897	0.893	ID	
[FJ787304] *Parafilaroides gymnurus*	0.635	0.633	0.638	0.638	0.638	0.638	0.638	0.633	0.599	ID

One slide for each case that was identified as positive both during histology and following sequencing for *Parafilaroides* sp. was selected for immunohistochemical analysis for the detection of *Brucella* spp. associated with parasites. The detection system used was the universal polymer bound to horseradish (MACH-A Universal HRP-Polymer, Biocare, Pacheco, USA) and the polyclonal anti-*Brucella abortus* primary antibody (non-commercial) produced in rabbits. Antigen retrieval was achieved with Proteinase K (Dakocytomation, Carpinteria, CA, USA) for 10 min and the reactions were revealed with chromogen 3-amino-9-ethylcarbazole (AEC Romulin; Biocare, Pacheco, USA). As a positive control, lung slides from a bovine fetus infected with *B. abortus* (Antoniassi *et al*., 2016) were used and negative controls were incubated with commercial universal serum (Universal Negative Control Serum; Biocare, Pacheco, USA).

## Results

Of all 26 fur seals of the species *A. australis* collected, 14 animals (53.8%) were classified as sub-adults, 8 as juveniles (30.8%) and 4 as adults (15.4%). Fifteen animals were males (57.7%) and 11 were females (42.3%). At necropsy, poor body condition was observed in 11 (42.3%) South American fur seals, good in 8 (30.7%) and moderate in 7 animals (26.9%). Gross and histological changes were analysed and the cause of death was determined for each fur seal when possible. Of these, 9 (34.6%) were diagnosed with traumas of different natures according to the macroscopic characteristics of the lesions – blunt trauma, puncture-cutting and blunt-blunt trauma (most frequent); 8 animals with poor body condition score and an empty stomach, sometimes containing liquid, were diagnosed with starvation (30.7%); and 9 were inconclusive (34.6%) due to unspecific morphological alterations (Table 2).

**Table 2.** Causes of death of South American fur seals infected by *Parafilaroides* sp., stranded in Southern Brazil

*Arctocephalus australis*	Body condition (score)	Cause of death	Metastrongyloidea histopathology (HE)	Species detected by PCR
1	Good	Cut-to-blunt trauma	+	
2	Bad	Starvation	–	
3	Moderate	Blunt polytrauma	–	
4	Good	Oesophageal rupture with secondary abscessive myositis (*Streptococcus* sp.)	+	*Parafilaroides* sp.
5	Good	Inconclusive	+	
6	Moderate	Blunt and sharp trauma	–	
7	Bad	Starvation	–	
8	Good	Blunt-cut trauma	–	
9	Moderate	Inconclusive	+	
10	Moderate	Inconclusive	+	*Parafilaroides* sp.
11	Good	Inconclusive	+	*Parafilaroides* sp.
12	Bad	Starvation	–	
13	Bad	Blunt-cut trauma	+	*Parafilaroides* sp.
14	Good	Blunt-cut trauma	+	
15	Moderate	Inconclusive	+	
16	Good	Inconclusive	–	
17	Good	Inconclusive	+	*Parafilaroides* sp.
18	Bad	Blunt-cut trauma	–	
19	Bad	Starvation	–	
20	Bad	Starvation	+	*Parafilaroides* sp.
21	Moderate	Blunt-cut trauma	–	
22	Bad	Starvation	–	
23	Moderate	Inconclusive	–	
24	Bad	Inconclusive	+	
25	Bad	Starvation	–	
26	Bad	Starvation	–	

**Fig. 1.**
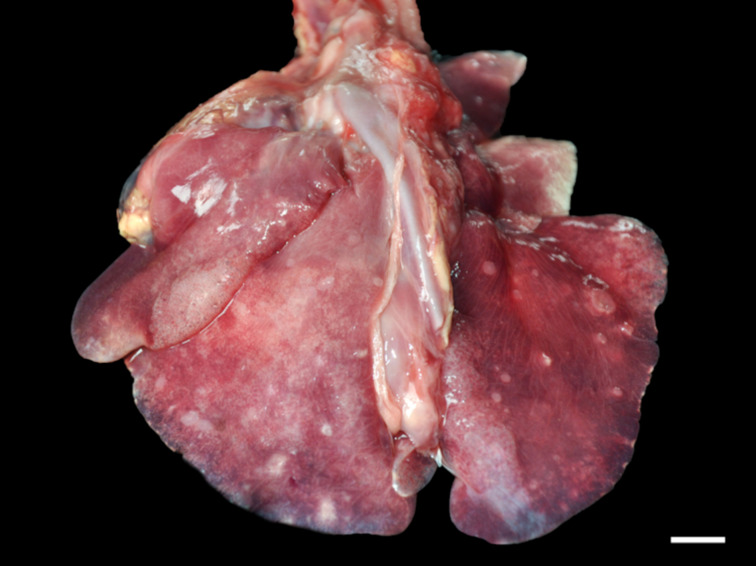
Verminotic pneumonia by *Parafilaroides* sp. in the South American fur seal. Multiple, whitish nodules measuring 0.5–2.5 cm in the lung lobes (3 cm bar).

Grossly, only 1 fur seal infected by *Parafilaroides* sp. (1/26; 4%) presented pulmonary alterations in all lung lobes, characterized by a moderate amount of circumscribed, white and sub-pleural nodular areas, with slight elevation, ranging from 0.5 to 2.5 cm in diameter, in addition to moderate diffuse oedema and multifocal to coalescing emphysema (Fig. 1). In another 14 animals analysed, which were later diagnosed with parafilaroidiasis by histology or PCR, lesions were interpreted as non-specific, such as pulmonary oedema, multifocal emphysema and small foci of atelectasis.

**Fig. 2.**
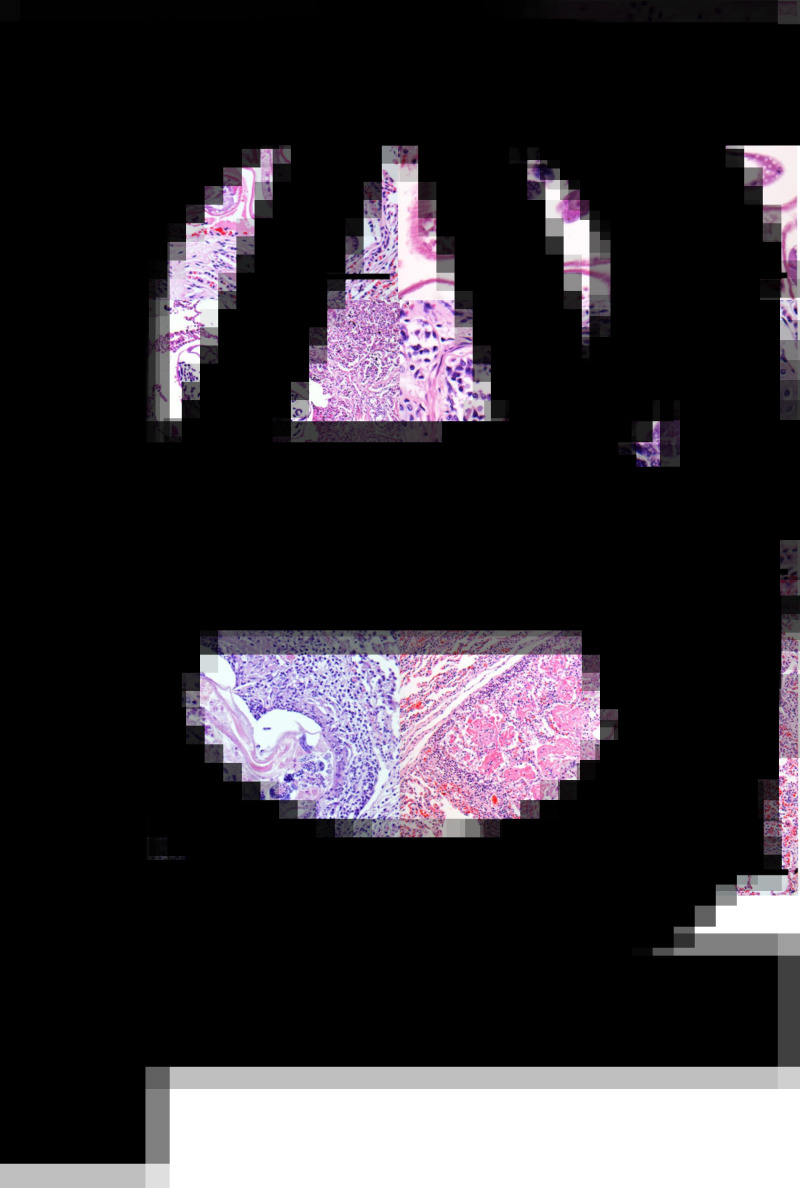
Histology of verminotic pneumonia caused by *Parafilaroides* sp. in the South American fur seal. (A) Metastrongylids composed of a thin eosinophilic cuticle; a thin hypodermis where the lateral cords are inserted (arrows) is observed. In the pseudocoelomic cavity, there are several sections of intestine composed of a single cell layer (asterisks) (50 *μ*m bar), haematoxylin and eosin (H&E) stain. (B) Female nematode containing larvae eggs inside the uterus and sperm deposit (asterisk) (20 *μ*m bar), H&E stain. (C) Cross-sections of female nematode parasites with intrauterine larval eggs are observed, organized in groups and located in the alveolar spaces, generating marked alveolar emphysema with the absence of an inflammatory infiltrate. Adjacent to the emphysematous areas, the parenchyma is moderately atelectatic (100 *μ*m bar), H&E stain. (D) Bronchiole lumen with nematode cross-section. Female, with a female reproductive system and eggs hatched inside the uterus (20 *μ*m bar), H&E stain. (E) Degenerated parasitic sections, with and without larval eggs, and surrounded by mononuclear inflammatory cells and fibrin (50 *μ*m bar), H&E stain. (F) Nodular lesion with a granulomatous appearance, characterized by eosinophilic fibrillar material, associated with a mononuclear inflammatory infiltrate and cellular debris, and surrounded by fibrous connective tissue (100 *μ*m bar), H&E stain.

With respect to the histology results, the pulmonary alterations found in the 12 animals, related to verminotic pneumonia, were classified as mild or moderate according to their extension and severity. No animal was diagnosed with severe verminotic pneumonia, and the individuals identified with the most severe conditions were associated with the most necrotic, inflammatory lesions, predominantly formed by granulomas randomly distributed in the lung parenchyma. In 46.1% (12/26) of the South American fur seals, transverse and longitudinal sections of nematodes were observed, ranging from 30 to 130 *μ*m. Groups of 2–8 parasites were observed that exhibited a thin eosinophilic cuticle, pseudocoelomatic cavity, polymyar/coelomian musculature, thin hypodermis, lateral cords and an evident digestive system (Fig. 2A). They were dioecious, with the male being smaller and less observed than females. The females had ovaries, eggs (Fig. 2B) and a uterus with or without larval eggs. Sperm deposits were sometimes observed in females (Fig. 2B).

The parasites were predominately found in the alveoli, comprising 75% (9/12) of the fur seals that were positive on microscopy. They were mostly seen in the periphery of the lung lobes, close to the visceral pleura (sub-pleural) or, less commonly, with a random multifocal distribution. Of the 9 cases in which parasites were seen in the alveoli, 5 were found exclusively in the alveolar lumen, while in 4 cases, there were also parasitic cuts in the bronchi or bronchioles. In 16.7% (2/12) of cases, parasites were seen in the bronchial space, and in 41.7% (5/12) they were identified in the bronchiolar space. Numerous emphysematous areas of variable extent, generally related to the number of nematodes (Fig. 2C), were associated with the luminal location of the parasites. Obstructive pulmonary atelectasis was frequently observed in these cases, and they were mainly adjacent to the areas of emphysema. The presence of the parasite inside the bronchi and bronchioles also caused alveolar pulmonary emphysema, and also led to the accumulation of a discrete amount of mucinous material in the lumen. In 33.3% of the animals (4/12), multifocal circumscribed areas composed of epithelioid and foamy macrophage infiltrates, and a smaller amount of eosinophils and fibrin, were observed, which involved parasites in different stages of degeneration (Fig. 2E). Externally to these structures, there was a moderate infiltrate of lymphocytes and plasma cells, surrounded by bundles of collagenous connective tissue (Fig. 2F). In the other 8 cases (66.7%), there was a slight interstitial infiltrate of lymphocytes around the adult nematodes. Of these animals with mild-to-moderate inflammation associated with these nematodes, different areas in the lung parenchyma with groups of parasites without an associated inflammatory reaction were frequent, comprising 66.6% of cases (8/12). Multifocal and moderate pulmonary arterial thrombosis was observed in 2 parasitized animals (2/12; 16.7%). Circulatory changes, such as congestion and diffuse alveolar oedema, were frequently noted in the lungs of these infected animals.

Of the 26 lung samples tested during PCR with *ITS-2*, 9 had bands of the expected size. The 6 sequenced samples (selected because they were also positive during histology) showed sequences compatible with *Parafilaroides* sp. registered in GenBank with accession numbers KP402084 and KP402085, by Jacobus *et al*. (2016), with a percentage similarity ranging from 99.82 to 100%. The sequences were deposited in GenBank under the following accession numbers: ON059340, ON059341, ON059342, ON059343, ON059344 and ON059345. The respective amplified base pairs are specified in Table 3.

**Table 3.** GenBank BLAST and results for the *ITS-2* region in *Parafilaroides* sp.

Sample	Fragment size (bp)	BLAST similarity percentage	Coverage percentage (%)	GenBank access number
*Parafilaroides* sp. (1)	568	99.82% com *Parafilaroides* sp. *Genbank*: (KP402084)	99	ON059340
*Parafilaroides* sp. (2)	623	99.65% com *Parafilaroides* sp. *Genbank*: (KP402084)	90	ON059341
*Parafilaroides* sp. (3)	648	99.82% com *Parafilaroides* sp. *Genbank*: (KP402085)	87	ON059342
*Parafilaroides* sp. (4)	578	99.27% com *Parafilaroides* sp. *Genbank*: (KP402084)	94	ON059343
*Parafilaroides* sp. (5)	576	100% com *Parafilaroides* sp. *Genbank*: (KP402084)	92	ON059344
*Parafilaroides* sp. (6)	586	99.65% com *Parafilaroides* sp. *Genbank*: (KP402084)	96	ON059345

**Fig. 3.**
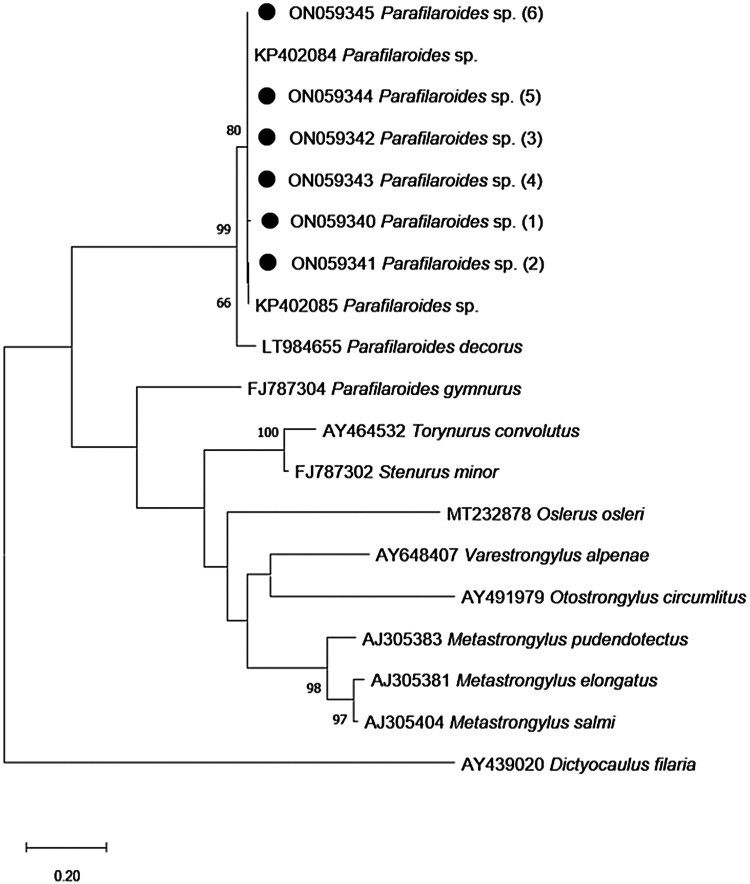
Maximum-likelihood phylogenetic tree 2. Performed with the Hasegawa**–**Kishino**–**Yano model with a gamma distribution (+G) of partial sequences of the *ITS-2* gene. The numbers in the branches indicate bootstrap values. Only bootstrap values >50 are counters. The numbers displayed next to the species name are the GenBank^®^ accession numbers of the sequences used. The generated sequences for the *Parafilaroides* sp. are in bold, indicated by a black circle.

The phylogenetic tree that was developed in this study demonstrates a close genetic relationship with the species of *Parafilaroides* spp. (Fig. 3), forming a clade with the sequences KP402084 and KP402085 (Jacobus *et al*., 2016) with 80% bootstrap. In addition, with 99% bootstrap, a macroclade including the species *P. decorus* that also parasitizes on pinnipeds was formed.

The 6 individuals with confirmed parafilaroidiasis at PCR and histology had their lungs submitted for an immunohistochemical test to detect anti-*B. abortus*, and the results were negative (absence of immunostaining).

## Discussion

The diagnosis of *Parafilaroides* sp. in the South American fur seals from the present study was established by histological analysis, PCR and sequencing. At necropsy, *Parafilaroides* spp. are described as small and delicate parasites (Jacobus *et al*., 2016) and are responsible for the formation of parasitic granulomas commonly described in pinnipeds (Measures, 2001; Reisfeld *et al*., 2019). However, such lung injury was observed in only 1 animal in the present study. Another macroscopic presentation characterized by the formation of small, yellowish pustules, which contained parasites inside them, was described in pinnipeds parasitized by *Parafilaroides* spp. (Dailey, 1970; Echenique *et al*., 2020). According to Dailey (1970), such a lesion was visualized when the infection was massive, and these small, firm nodules were associated with the formation of parasitic granulomas in degenerated specimens. This type of inflammatory reaction would occur in animals that are more susceptible to infections, such as in debilitated individuals and in stressful situations (Measures, 2001). As in the present study, most cases of pulmonary verminosis in fur seals were not diagnosed macroscopically (Onderka, 1989; Measures, 2001).

The localization and histological characterization of the identified pulmonary parasites was compatible with the Metastrongyloidea Superfamily (Bowman, 2014). The 2 pulmonary nematodes described in otarids, *O. circumlitus* and *Parafilaroides* spp., are mainly distinguished based on the characteristics inherent to their respective families, in which Crematosotidae has a bursa, while in Filaroididae, it is absent or indistinct, observed by the parasitic morphology that unfortunately we were unable to perform due to the absence of adult worms for analysis. *Otostrongylus circumlitus* is larger and therefore easily identified on macroscopy, in addition to being located in the bronchi, bronchioles and occasionally in the arteries. On the other hand, in *Parafilaroides* spp., it is smaller and predominantly alveolar in location (Onderka, 1989; Kelly *et al*., 2005). Microscopically, parasitic size may help suggest metastrongylid genus; however, in this study only the molecular tool concluded the diagnosis. *Otostrongylus circumlitus* presents with an approximately 900 *μ*m, with thick cuticle and hypodermis (Barnett *et al*., 2019), while in *Parafilaroides* spp., as seen in the present work, they have a diameter ranging from 30 to 130 *μ*m, and a smooth and thin cuticle (Garner *et al*., 1997).

*Parafilaroides* spp. cause respiratory disease in pinnipeds and can result in high mortality rates (Dailey, 1970; Onderka, 1989; Garner *et al*., 1997; Siebert *et al*., 2007). However, in South American fur seals, infection by these agents usually causes mild-to-moderate lung lesions, possibly suggesting a strategic co-evolution process to avoid host loss (Onderka, 1989; Sukhdeo, 1994). Although areas of atelectasis and emphysema in the lungs were observed in the present study, they were associated with a slight reduction in the respiratory parenchyma. Furthermore, the inflammatory process was more severe and granulomatous when there were degenerated parasitic structures, similarly seen by Onderka (1989). Although such pulmonary worm infections have not been considered serious enough to lead to death by themselves, they are associated with some degree of debilitation in these individuals, interfering with their feeding behaviour, their ability to escape from predators and threats, and to rest, even favouring the emergence of secondary infections (Measures, 2001). Two animals infected by *Parafilaroides* sp. died from traumatic causes, and 1 from starvation. These frequent causes of death in South American fur seals were also reported by other authors (Seguel *et al*., 2011, 2013; Katz *et al*., 2012; Baldassin *et al*., 2017). In Brazil and other South American countries (outside the breeding colonies), traumatic causes are related to the interaction of these animals with dogs, and to human interactions, either when fishing or due to aggression (Katz *et al*., 2012; Baldassin *et al*., 2017). Of the 9 cases diagnosed as trauma in this study, 7 were characterized in juvenile or sub-adult animals as resulting from blunt force trauma with frequent perforations in the skin and lacerations of muscle and adjacent tissues, which resembled a dog bite. The animals in this study, whose final diagnosis was determined to be starvation, and who also had verminotic pneumonia, did not present with any lung lesions that were severe enough to justify this as a cause of death. This is unlike what was found in the sub-Antarctic fur seal, as reported in SC state by Reisfeld *et al*. (2019) which, despite being cachectic and carrying infections by *Sarcocystis* sp. and gammaherpesviruses, had pneumonia caused by *Parafilaroides* sp. and was suggested as the cause of death, determined by the most severe lesion.

Studies often describe pinnipeds parasitized by *Parafilaroides* spp. and these are affected by other aetiological agents. It is further suggested that this nematode is a host for *Brucella pinnipedialis* (Rhyan *et al*., 2018; Reisfeld *et al*., 2019). However, the South American fur seals studied by our team did not show immunohistochemical staining with anti-*B. abortus* polyclonal antibody (Rhyan *et al*., 2018), suggesting that lung nematodes in this region are not related to this bacterial species or to other species of the same genus, possibly due to the distance between the reproductive colonies and the places in which these animals were found.

Apparently, the species of *Parafilaroides* have higher occurrences in certain regions, according to the literature already described (Measures, 2001; Dailey, 2009; Echenique *et al*., 2020). In this way, although in this present study the pulmonary nematodes were concluded only at the gender level, it is noted that the descriptions in fur seals from the Southern Hemisphere were more frequently related to the species *P. normani* (Dailey, 2009; Jacobus *et al*., 2016; Echenique *et al*., 2020). In Brazil, previous reports suggest (Jacobus *et al*., 2016) or affirm (Echenique *et al*., 2020) that the species of lung worms that affected fur seals was *P. normani*.

In addition to the fact that the morphology observed in the histological analyses was consistent with the genus *Parafilaroides*, the phylogenetic analysis results showed high similarity with the sequences KP402084 and KP402085 with respect to *ITS-2*, which was deposited as *Parafilaroides* sp. by Jacobus *et al*. (2016). Although the authors deposited the *ITS-2* sequences as *Parafilaroides* sp. when they sequenced a fragment of the cytochrome c oxidase subunit I mitochondrial DNA gene from one of their samples, it showed 100% similarity with *P. normani* (Jacobus *et al*., 2016). Since the sequences of the present study present high similarity with those of the study by Jacobus *et al*. (2016), as demonstrated in the identity matrix, it is highly likely that the species involved in the pulmonary verminosis of the specimens studied here is also *P. normani.* Furthermore, the monophyletic relationship of the sequences from the present study with the sequences produced by Jacobus *et al*. (2016), as well as the high bootstrap value (80%) of the branch in question and the percentage of similarity shown in the matrix, reaffirms the possibility that they are the same species. When compared to the molecular test, the morphological evaluation proved to be superior for the detection of pulmonary parasites. Since the fragments were randomly removed from different areas, the difference in analysis results was possibly due to the multifocal nature of the lesion, which was associated with the greater volume of the lung tissue analysed during histology. The 25 mg scanty fragments used for DNA extraction may not contain the parasite. In the same way, we obtained 3 positive results during the molecular analysis of animals that histologically did not present parasitic structures, possibly also due to the random selection of tissues for the different techniques. However, the molecular examination proved to be an excellent complementary tool, not only because of its high specificity, but also because it constitutes another form of diagnostic screening. Furthermore, the results of the PCR products for the *ITS-2* gene were satisfactory for sequencing and phylogenetic analysis.

Metastrongylids verminotic pneumonia was morphologically observed in the studied South American fur seals, and the lesions featured a mild-to-moderate morphological pattern. The characteristic macroscopy results of the lesion by *Parafilaroides* spp. were evidenced in 1 animal, which presented with a nodular, multifocal and moderate lesion due to the formation of granulomas associated with the degenerated parasite. The histology results established the diagnosis, characterized the lung lesion and determined its intensity. Furthermore, PCR, with the amplification of a fragment of the *ITS-2* gene, confirmed that the genus was involved in verminotic pneumonia. Combined, these approaches made it possible to suggest that the species involved in this infection is *P. normani.*

## Data Availability

The data that support the findings of this study are available from the authors upon reasonable request.
